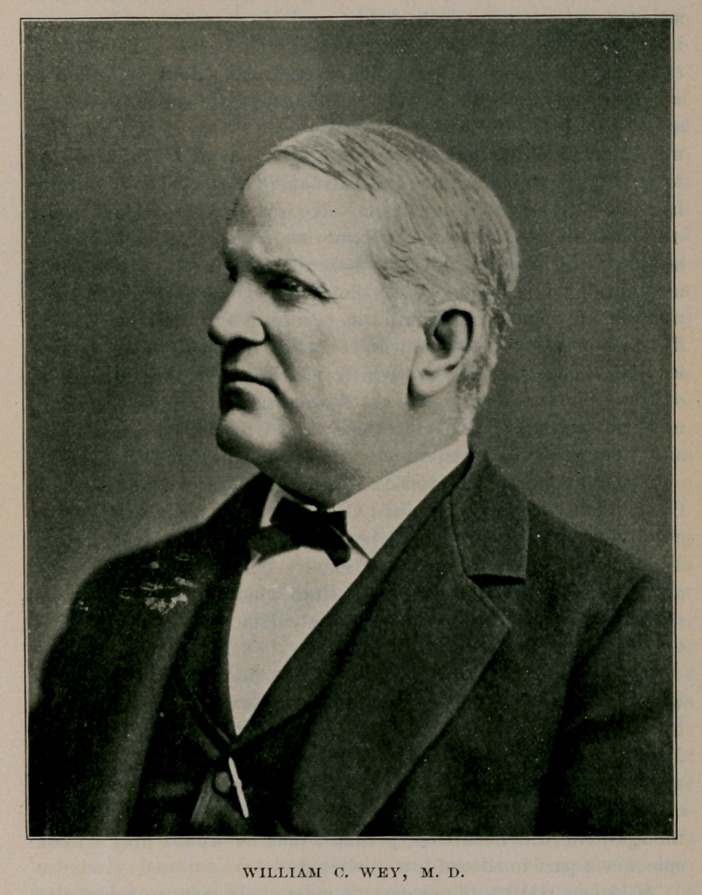# Dr. William C. Wey

**Published:** 1897-08

**Authors:** 


### Obituary.

Dr. William C. Wey, of Elmira, died at his residence in that city July 1, 1897, aged 68 years. Though he had been in failing health for some months his end came somewhat unexpectedly, yet serenely peaceful. Dr. Wey was born at Catskill, January 12, 1829. His father, William H. Wey, was a druggist, and his great-grandfather, Dr. Thomas O. H. Croswell, was a practising physician of note in the village. In his student days he became a pupil of Dr. Alden March, whose surgical assistant he was, and he graduated from the Albany Medical College January 23, 1849. He located at Elmira in the spring of the same year, which was before the village enjoyed railroad facilities, so that in the early years of his practice Dr. Wey led the life of the country doctor of the period.

He soon acquired a surgical reputation and went long distances into the country to perform difficult operations in the pre-anesthetic period.

WILLIAM C. WEY, M. D.

Early in the war of the rebellion Dr. Wey was appointed an examiner of recruits, later he was appointed by the United States government surgeon-in-charge of the hospital, and still later, medical officer to the confederate prison at Elmira. In his early professional career he served as coroner of Chemung county, and was the first health officer in Elmira after it became a city; again in 1894 he was appointed health officer, serving in that capacity until failing health compelled him to relinquish his duties. For six years he was a member of the board of education, taking great interest in the affairs of the board, and, it is needless to add, discharging the duties thereof with energy and fidelity. Dr. Wey was appointed, by Governor Tilden, a manager of the State reformatory, March 5, 1875, and he served continuously in this capacity until his death, a period of over twenty-two years. He was also, for a number of years, one of the managers of the New York State Inebriate Asylum at Binghamton. He was a manager and senior consultant of the Arnot-Ogden Memorial Hospital, and the last time he left his residence, a fortnight before his death, was to attend a meeting of the managers of the hospital. During the forty-eight years of his residence in Elmira he occupied but three offices: the first was in the old Dunn Block, corner of Lake and Water streets, where he remained until the winter of 1853; he then moved his office to a house situated on East Water street, where he remained until the autumn of 1876; he then moved to the corner of Main and West Second streets, which office he occupied until his end.

Dr. Wey was married, November 15, 1853, to Mary Bowman Covell, daughter of Dr. Edward Covell, of Wilkesbarre, Pa. Two children were born to them, both now living—Dr. Hamilton D. Wey and Mrs. Ray Tompkins, of Elmira. Mrs. Wey died in 1877, and his daughter married in 1893, since which time the father and son alone occupied the family home. His sister, Mrs. Seth H. Grosvenor, of Buffalo, also survives.

Dr. Wey was elected president of the Medical Society of the State of New York in 1871, presiding at the meeting held the following year. He chose for the subject of his presidential address, Medical responsibility and malpractice. It was a scholarly paper, attracted considerable attention, and evinced much knowledge of forensic medicine. His closing words were prophetic, though little did he dream, perhaps, that he would play so conspicuous a part in the reform predicted.

Said he: "With the profession rests the power to accomplish a reform of the system of medical education; such a thorough reform as will extend throughout the nation. Some among you, I know, have pictured to yourselves an ideal standard of preparation for the profession, including the processes by which a pupil is carried through the stages of growth in medicine to the possession of his degree, and so on to the higher positions to which his qualifications give him access. Through much strife, opposition and discouragement such a change is surely coming. Not in your day, nor in mine, but in that future which awaits the greater development of our country, disenthralled medical science will stand conspicuously forth as teaching given from God."

Dr. Wey was chairman of the special committee on the code of ethics of the Medical Society of the State of New York, appointed by President Bailey in 1881, his colleagues being Drs. Agnew, Vander Poel, Ely and Piffard. This committee presented a report in 1882, revising the code of ethics of the American Medical Association that had heretofore been in force in the state medical society. An exciting debate ensued, a substitute having been presented by Dr. Roosa providing for the abolishment of all written codes. Finally, however, the new code was adopted by a two-thirds' vote, this action being ratified in 1883, and again in 1884. As a consequence the Medical Society of the State of New York was disbarred from representation in the American Medical Association, a condition that has continued to the present time. Subsequently the state medical society abolished all written codes and is now governed by the only rule that obtains among gentlemen —that of honor.

Dr. Wey was chosen president of the State Medical Examining and Licensing Board at its first meeting, September 1, 1891, in which place he continued until his death. He was also examiner in physiology and hygiene, and through his scholarly attainments, interest in the work, and efficiency as an examiner and presiding officer, contributed largely to the success attained by the state medical examining board in elevating the status of the profession of medicine.

Dr. Wey was a man possessed of great dignity of character, rare courtliness of manner, and was a representative of all that goes to make up the best type of nature's noblemen. In his chosen profession he occupied conspicuous eminence, which, through his scholarly learning, marked ability and wealth of resource, contributed to make him easily the most eminent physician in the Chemung Valley, a statement that need in nowise disparage his colleagues. Above and beyond his profession he was a man of accomplishment, well trained to thought and action, a resourceful debater and an easy speaker, a wit at the dinner table, possessed of a musical voice, a winsome manner and a sweetness of expression that made him socially one of the most attractive of men. It will not be easy to fill his place in the several official positions that he so ably and gracefully adorned, nor in social, professional or civil life—in all of which he leaves a cherished memory and an inspiring example.

His obsequies were simple, only the ritual of the Episcopal church, and that was all. His remains were followed to their last resting place by the family, the Medical Society of the County of Chemung, the pall-bearers, and the State Medical Examining Board, the members of which latter body came from the four corners of the state to pay a last tribute of respect to the memory of their beloved colleague.

## Figures and Tables

**Figure f1:**